# Regulation of p53 during senescence in normal human keratinocytes

**DOI:** 10.1111/acel.12364

**Published:** 2015-07-01

**Authors:** Reuben H Kim, Mo K Kang, Terresa Kim, Paul Yang, Susan Bae, Drake W Williams, Samantha Phung, Ki-Hyuk Shin, Christine Hong, No-Hee Park

**Affiliations:** 1 UCLA School of DentistryLos Angeles, CA, 90095, USA; 2 UCLA Jonsson Comprehensive Cancer CenterLos Angeles, CA, 90095, USA; 3 UCLA David Geffen School of MedicineLos Angeles, CA, 90095, USA

**Keywords:** aging, cancer, epigenetics, human keratinocytes, p53, replicative senescence

## Abstract

p53, the guardian of the genome, is a tumor suppressor protein and critical for the genomic integrity of the cells. Many studies have shown that intracellular level of p53 is enhanced during replicative senescence in normal fibroblasts, and the enhanced level of p53 is viewed as the cause of senescence. Here, we report that, unlike in normal fibroblasts, the level of intracellular p53 reduces during replicative senescence and oncogene-induced senescence (OIS) in normal human keratinocytes (NHKs). We found that the intracellular p53 level was also decreased in age-dependent manner in normal human epithelial tissues. Senescent NHKs exhibited an enhanced level of p16^INK4A^, induced G_2_ cell cycle arrest, and lowered the p53 expression and transactivation activity. We found that low level of p53 in senescent NHKs was due to reduced transcription of p53. The methylation status at the p53 promoter was not altered during senescence, but senescent NHKs exhibited notably lower level of acetylated histone 3 (H3) at the p53 promoter in comparison with rapidly proliferating cells. Moreover, p53 knockdown in rapidly proliferating NHKs resulted in the disruption of fidelity in repaired DNA. Taken together, our study demonstrates that p53 level is diminished during replicative senescence and OIS and that such diminution is associated with H3 deacetylation at the p53 promoter. The reduced intracellular p53 level in keratinocytes of the elderly could be a contributing factor for more frequent development of epithelial cancer in the elderly because of the loss of genomic integrity of cells.

## Introduction

Cancer is one of the most prevalent age-associated diseases in elderly populations and is one of the leading causes of death, second to cardiovascular diseases (Balducci & Beghé, 2002). Age-dependent increase in cancer incidence is largely attributed to an increased susceptibility to environmental carcinogens as age-related molecular changes occur over time in cells. In particular, the loss of tumor suppressive functions in cells plays a key role, which would result in the cells having accumulated genetic mutations over time, ultimately leading to aberrant cell growth and cancer development (Hinkal & Donehower, 2007).

p53 is a potent tumor suppressor and known as ‘the guardian of the genome’ due to its involvement in a variety of cellular mechanisms including DNA repair, apoptosis, cell cycle arrest, and senescence (Kruse & Gu, 2009). The tumor suppressive role of p53 in the context of aging is clearly demonstrated *in vivo*. In a transgenic mouse model, a truncated form of p53 that augments wild-type p53 activity enhanced resistance to spontaneous tumor development; however, these mice exhibited premature aging phenotypes (Tyner *et al*., 2002). On the other hand, another group showed that the ‘super p53’ mice, which harbor an extra copy of p53 transgene under its natural promoter, protected the animals from cancer without premature aging (García-Cao *et al*., 2002). Recent reports showed that the expression of p53 declines in neural progenitor cells and mesenchymal stem cells in an age-dependent manner (Mikheev *et al*., 2009; Wilson *et al*., 2010) and that p53 activity decreases as a function of age at the organismal level (Feng *et al*., 2007). Collectively, these findings suggest that the loss of p53 expression during aging may mediate the development of cancer in an aging-dependent manner.

Similar to multicellular organisms, normal cells *in vitro* undergo limited replicative lifespan called ‘replicative senescence’, and p53 is implicated in this *in vitro* aging process. However, the role of p53 during organismal aging and replicative senescence seems to be incongruent. Under normal conditions in healthy and unstressed cells, p53 protein level is known to be very low or undetectable due to its short half-life mediated by its interaction with MDM2 (Gudkov & Komarova, 2007; Donehower, 2009; Lee & Gu, 2010). During replicative senescence, p53 expression level is reported to be similar in young and senescent fibroblasts (Atadja *et al*., 1995), while others report that its expression level increases in human diploid fibroblasts (Kulju & Lehman, 1995). Increase in the transcriptional activity of p53 has also been reported during replicative senescence in human fibroblasts (Vaziri *et al*., 1997).

Although the role of p53 during replicative senescence in normal cells *in vitro* was well characterized, most of these studies were carried out in normal human fibroblasts (NHFs) whose molecular characteristics and behaviors are notably different from those of normal human keratinocytes (NHKs). Compared to NHFs, the expression level of p53 is significantly higher in actively proliferating NHKs. Previous studies reported that high level of p53 was progressively decreased during the replicative senescence in NHKs (Kim *et al*., 2010; Sisoula *et al*., 2010; Wilson *et al*., 2010), indicating that p53 may not be directly associated with the replicative senescence in NHKs. These observations prompted us to revisit and investigate the status of p53 during replicative senescence in NHKs.

Here, we report that p53 level diminishes during the replicative senescence and OIS at the transcriptional level, and this diminution of p53 transcript is associated with H3 deacetylation at the p53 promoter. Our study suggests that the reduced intracellular p53 level in keratinocytes of the elderly could be a contributing factor for more frequent development of epithelial cancer in the elderly because of the loss of genomic integrity of cells.

## Results

### Expression and transactivation activity of p53 diminishes during replicative senescence in NHKs

**Fig 1:**
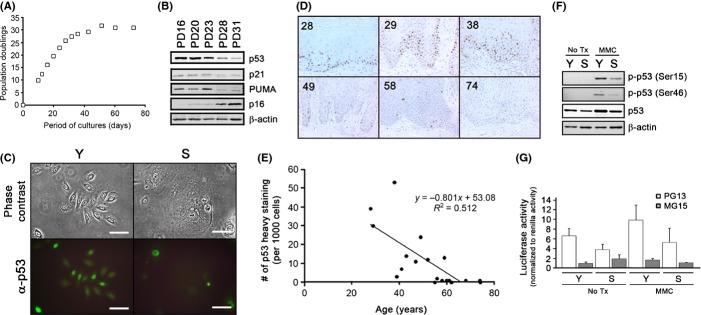
p53 protein expression progressively decreases in normal human keratinocytes (NHKs) and normal human oral epithelia (NHOE) during replicative senescence of *in vitro* and organismal aging *in vivo*. (A) The primary NHKs were serially subcultured until they senesced, and the population doubling curve was obtained. (B) NHKs at the different PDs were subjected to Western blotting for the expression of p53, p21, PUMA, and p16. β-Actin was used as a loading control. (C) Immunofluorescent staining was performed in young (Y) and senescent (S) NHKs using IgG (Not shown) or p53 antibody (DO-1) at 1:1000. The bar represents 100 μm. (D) Normal human oral epithelium (NHOE) from 20 healthy individuals were formalin-fixed, paraffin-embedded, sectioned, and subjected to immunohistochemical analysis for the expression of p53 (DO-1) at 1:100 concentration. The numbers at the upper-left corner of each photograph (100×) represent the age of the donors. (E) The representative field was randomly selected by a third person, and one thousand cells were counted for heavy staining (> 80%), moderate staining (40–80%), mild staining (5–40%), or no staining (< 5%). Only the heavy-staining cells were counted and plotted against the age. Linear regression analysis was performed (*R*^2^ = 0.512). (F) Young and senescent NHKs were treated with mitomycin C (MMC) (10 μm) and subjected to Western blotting for phosphorylated p53 at ser15 and ser46 residues. (G) Young and senescent NHKs were transfected with PG13- and MG15-luciferase reporter plasmids, treated with MMC (10 μm), and luciferase activities were measured. pRL-SV40 plasmid were cotransfected to normalize the luciferase signals. The experiments were triplicated.

It has been shown that p53 protein expression and/or transactivation activity in NHFs increases during senescence (Atadja *et al*., 1995; Kulju & Lehman, 1995; Vaziri *et al*., 1997). Consistent with previous reports, we found that p53 expression was low or undetectable in actively proliferating NHFs, but the expression increased during replicative senescence (Fig. S1; Supporting information). On the other hand, others and we reported that p53 protein level diminishes during senescence in NHKs (Kim *et al*., 2010; Sisoula *et al*., 2010; Wilson *et al*., 2010). To confirm this finding, we serially subcultured NHKs until they senesced (Fig.1A), and cells at different population doublings were harvested and subjected to Western blotting. As expected, p53 protein level decreased during replicative senescence in NHKs (Fig.1B,C). Similarly, the expressions of p53-targeting genes, p21 and PUMA, were also progressively diminished, whereas p16, a marker for senescence, gradually increased as cell senesced (Fig.1B). Significant amounts of p53 were also observed at the 0 passage of the primary NHKs, and the diminution of p53 during serial subculturing of NHKs was also detected using different clone of p53 antibody, indicating that such observations were independent of cell culture conditions nor antibodies (Fig. S2; Supporting information).

We questioned whether the diminution of p53 during senescence also occurs in the aging process *in vivo*. To address this question, we obtained normal human oral epithelia (NHOE) from 20 healthy individuals at different ages. These tissues were subjected to paraffin embedding, and immunohistochemical staining was performed against p53. Interestingly, we also found that the p53 protein expression decreased in an age-dependent manner (*r* = 0.512); high numbers of p53 positives were seen in the below 40s age group, a mixture of p53 staining patterns was observed in ages ranging from 40s to 60s, and almost a complete loss of p53 positives was observed in individuals in their 60s and above (Fig.1D,E). Our results suggest that the decrease in p53 protein expression also occurs *in vivo*.

To examine whether the p53 activation pathway remains intact during replicative senescence, we treated young and senescent NHKs with mitomycin C (MMC) and examined transactivation activity of p53 by screening the phosphorylation status of p53 and using a p53-responsive luciferase reporter assay. In MMC-treated NHKs, p53 became phosphorylated at Ser15 and Ser46 residues (Fig.1F), and the transactivation activity of p53 increased in both young and senescent NHKs to similar degree (Fig.1G) that is reflective of the basal p53 levels. These results suggest that p53 is functionally responsive to its activation pathways in spite of low amounts of p53 during senescence.

### NHKs undergo G_2_ cell cycle arrest during replicative senescence

**Fig 2:**
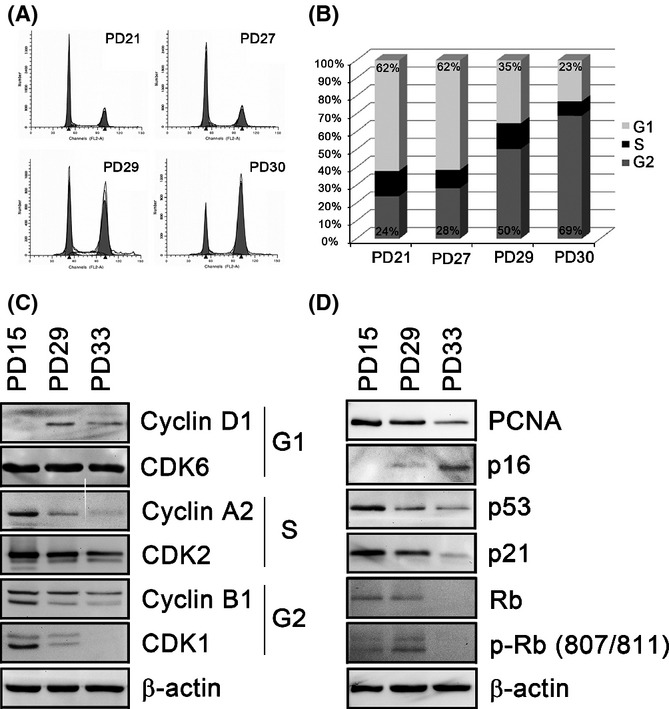
Normal human keratinocytes (NHKs) undergo G_2_ cell cycle arrest during replicative senescence. (A) NHKs at different PDs were analyzed for the cell cycle using flow cytometry. (B) The proportion of the cells in different cell cycle phases at different PDs was presented using a graph. (C) NHKs at early (PD15), middle (PD29), and senescent (PD33) stages were collected and subjected to Western blotting against cyclin D1, cyclin A2, cyclin G2, CDK6, CDK2, and CDK1. (D) The same NHKs at the same PDs were also subjected to Western blotting against PCNA, p16, p53, p21, Rb, and p-Rb (807/811). β-Actin was used as a loading control.

p53-mediated cell cycle arrest is known to occur from G_1_ to S phase (Dulić *et al*., 1993; Stein & Dulić, 1995), and senescent NHFs exhibit G_1_ cell cycle arrest (Afshari *et al*., 1993). Therefore, we performed cell cycle analysis to examine whether cell cycle arrest occurs independently of p53 during replicative senescence in NHKs. We found a progressive increase in G_2_-phase arrest as NHKs reach senescence (Fig.2A,B). When screened for cell cycle regulators, complete ablated expression of CDK1, a G_2_ cell cycle mediator, was notable (Fig.2C). Interestingly, the expression of another known tumor suppressor, pRb, was also diminished in senescent NHKs (Fig.2D). These data indicate that cell cycle arrest during replicative senescence in HNKs primarily occurs at the G_2_ phase.

### Diminution of p53 is not due to enhanced degradation of p53 during replicative senescence

**Fig 3:**
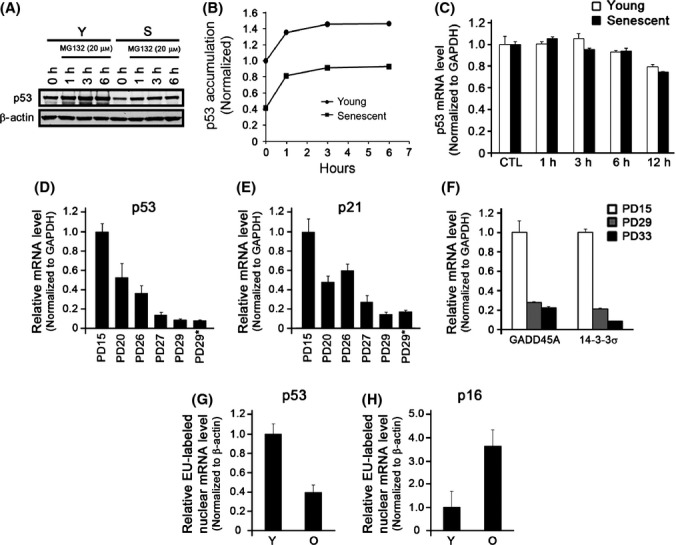
p53 is regulated at the transcriptional level. (A) Young and senescent normal human keratinocytes (NHKs) were treated with MG132 (20 μm) for 0, 1, 3, and 6 h. Cells were harvested and Western blotting was performed against p53 and β-Actin. (B) The densitometry analysis of (A) was performed to quantitate the amount of p53 using Image J. (C) Young and senescent NHKs were treated with Actinomycin D (2 μm) for 0, 1, 3, 6, and 12 h. Cells were harvested and qRT–PCR was performed for p53 expression. The expression of p53 mRNA for both young and senescent NHKs was normalized to those in untreated cells. The primary NHKs were serially subcultured until they senesced, and NHKs at the different PDs were subjected to qRT–PCR for the expressions of p53 (D) and p21 (E). GAPDH was used as a control. (F) The direct p53 target genes, GADD45A, and 14-3-3σ were evaluated using qRT–PCR. Newly synthesizing nascent p53 (G) and p16 (H) nuclear mRNAs were detected using Click-iT Nascent RNA Capture kit followed by qRT–PCR.

The level of p53 is known to be regulated mostly at the posttranslational level through protein modification preventing proteasome-dependent degradation, leading to stabilization and activation of p53 transactivation activity (Lee & Gu, 2010). Therefore, we reasoned that the diminution of p53 protein expression during senescence may be due to the enhanced degradation of p53. To test this hypothesis, we inhibited the proteasome-dependent degradation pathway and examined whether the level of p53 accumulates in senescent NHKs comparable to that in young NHKs. When young and senescent NHKs were treated with MG132 in a time-dependent manner, p53 protein level in both young and senescent NHKs reached their plateau levels as early as 3 h without further p53 accumulation in senescent NHKs, suggesting that the degree of accumulated p53 was similar (Fig.3A,B). Consistent with more increased level of transactivation activity and phosphorylation of p53 after MMC treatment in young NHKs as compared to senescent NHKs (Fig.1F,G), accumulated p53 after MG132 treatment induced apoptotic responses only in young NHKs (Fig. S3; Supporting information).

p53 is also regulated at the posttranscriptional level (e.g., mRNA stability) by numbers of proteins such as ribosomal protein L26 or HuR (Mazan-Mamczarz *et al*., 2003; Takagi *et al*., 2005). Therefore, we examined whether p53 mRNA stability was affected during replicative senescence in NHKs. We inhibited transcriptional machinery in young and senescent NHKs by treating cells with 2 μm actinomycin D and measured the p53 mRNA levels in a time-dependent manner. We found that the stability of p53 mRNA was similar in both young and senescent NHKs (Fig.3C). These data suggest that the diminished level of p53 during replicative senescence in NHKs is minimally affected at the posttranscriptional or posttranslational levels.

### Diminution of p53 during replicative senescence occurs at the transcriptional level

We next sought to examine mRNA levels of p53 and p21 using qRT–PCR. During replicative senescence, mRNA expressions of p53 and its target genes, p21, GADD45A, and 14-3-3σ, were all diminished notably (Fig.3D–F). To directly examine the decrease in p53 transcription, we measured the *de novo* p53 mRNA synthesis in young and senescent NHKs and found that p53 transcripts decrease, while p16 transcripts increase during replicative senescence (Fig.3G,H). The loss of p53 during replicative senescence at the transcriptional level did not seem to be dependent on culture condition (Fig. S4; Supporting information). Furthermore, the decreasing pattern of p53 mRNA and protein was also further confirmed using different primer sets and antibodies that target the different region of p53 mRNA and protein, respectively (Fig. S5; Supporting information). Collectively, our data indicate that expression of p53 diminishes at the transcriptional level during replicative senescence in NHKs.

### The status of DNA methylation and histone modifications at the p53 promoter

**Fig 4:**
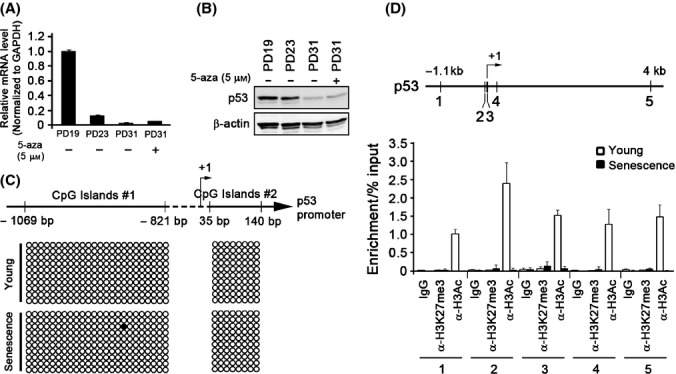
p53 is epigenetically regulated via histone deacetylation during replicative senescence. Senescent normal human keratinocytes (NHKs) were treated with 5-aza-CdR (5 μm) for 5 days. These cells, along with the same strain of NHKs at different PDs, were subjected to qRT–PCR (A) and Western blotting (B) to examine p53 expression. (C) Bisulfite sequencing assay was performed at two CpG islands in the p53 promoter regions in young and senescent NHKs. Ten samples were cloned and sequenced to confirm methylation status of the p53 promoter DNA at CpG islands #1 and #2. (D) The ChIP assay was performed in young and senescent NHKs using anti-H3Ac and anti-H3K27me3 antibodies, and the resultant DNA fragments were PCR-amplified using 5 different primers specific to the p53 promoter region (upper panel). The presence of p53 promoter DNA was evaluated using qRT–PCR (lower panel).

Recent studies showed that p53 transcription is also regulated by epigenetic mechanisms (Su *et al*., 2009; Soto-Reyes & Recillas-Targa, 2010). We thus examined the involvement of DNA methylation and histone modifications at p53 promoter region during replicative senescence in NHKs. First, to investigate whether global DNA demethylation would alter the expression of p53 in senescent NHKs, we exposed the cells to either 5-aza-CdR or zebularine, DNA-demethylating agents. Exposure of senescent NHKs to the above demethylating agents did not alter the levels of both p53 mRNA and protein in senescent NHKs (Figs4A,B, and S6; Supporting information). Moreover, we did not find any notable alterations in methylation status in the CpG islands of senescent NHKs as determined by bisulfite sequencing assay (Fig.4C). Next, to examine the involvement of chromatin modifications on p53 expression in senescent NHKs, we conducted chromatin immunoprecipitation (ChIP) assay using antibodies against acetylated histone 3 (H3Ac), an active mark, and tri-methylated histone 3 at lysine 27 (H3K27me3), a repressive mark. Higher level of H3Ac was abundantly noticed at the p53 promoter region in rapidly proliferating NHKs, while we detect negligible amount of H3Ac in senescent NHKs (Fig.4D), indicating that histone deacetylation likely contributes to the diminished expression of p53 during replicative senescence.

### The loss of p53 enhanced mutational frequency in NHKs

**Fig 5:**
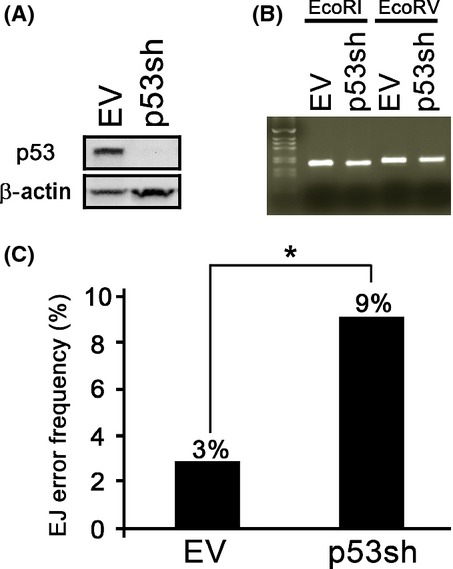
The loss of p53 enhances mutational frequency in normal human keratinocytes (NHKs). (A) NHKs infected with retrovirus expressing empty vector (EV) or p53shRNA (p53sh) were selected using puromycin (1 μg mL^−1^) and maintained. p53 expression was observed using Western blotting. (B) *In vitro* end joining assay was performed by incubating the lysates from these cells with ECoRI-or ECoRV- linearized pCR2.1-TOPO plasmid. The resultant reaction was subjected to PCR amplification with M13 primers to examine ligation efficiency. (C) Amplified PCR products from the ECoRI linearized and ligated pCR2.1-TOPO plasmid were subcloned and introduced into TOP10 cells. Subsequently, the single colony from a bacto-agar plate was subjected to PCR and sequencing analysis to examine errors in DNA. * *p* < 0.05.

We previously reported that senescent NHKs showed abnormal DNA end joining activity by increasing the frequency of end joining errors, which might contribute to genetic instability in the aging process (Kang *et al*., 2005). To examine whether the diminution of p53 during replicative senescence is directly associated with reduced DNA end joining activities in senescent cells, we knocked down p53 in rapidly proliferating NHKs (Fig.5A) and determined the *in vitro* DNA end joining assay capabilities. We found that the end joining capabilities of EcoRI- or EcoRV-linearized exogenous plasmids were similar in both p53 knockdown NHKs and the control counterpart (Fig.5B). However, when ligated plasmids were sequenced, there were significantly higher joining errors with mutated sequences in cells with p53 knockdown compared to the control (9% vs. 3%, *p* < 0.05; Fig.5C). The similar results were obtained using different p53 shRNA targeting different sequences (Data not shown). These data indicate that the loss of p53 is directly associated with the induction of DNA repair errors in NHKs.

### p53 expression decreases during OIS via H3 deacetylation

**Fig 6:**
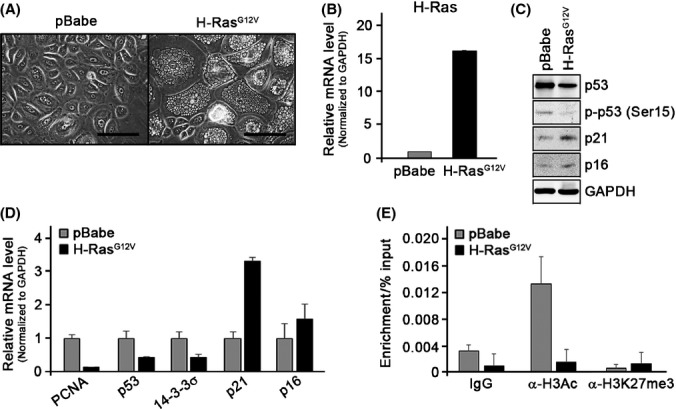
p53 expression decreases during oncogene-induced premature senescence via H3 deacetylation. (A) Actively proliferating normal human keratinocytes (NHKs) were infected with retrovirus expressing empty vector (pBabe) or H-Ras^G12V^ and selected using puromycin (1 μg mL^−1^). The bar represents 100 μm. (B) Cells were collected 6 days after infection, and overexpressed H-Ras mRNA levels were confirmed using qRT–PCR. Western blotting (C) and qRT–PCR (D) were performed on these cells. (E) ChIP assay was performed in NHKs with pBabe or H-Ras^G12V^ using anti-H3Ac and anti-H3K27me3 antibodies.

To investigate that the alteration of p53 level and its transactivation activity is truly due to senescence of NHKs, we also conducted similar study in NHKs senesced with H-Ras oncogene (Serrano *et al*., 1997). An ectopic overexpression of NHKs with H-Ras^G12V^ induced senescence of NHKs with flattening phenotypes and vacuolization in the cytoplasm (NHK/H-Ras^G12V^) (Fig.6A,B). NHK/H-Ras^G12V^ cells, similar to replicative senescent NHKs, exhibited lower level of intracellular p53 and higher level of p16 (Fig.6C,D). Interestingly, although the mRNA expression of p53 target gene 14-3-3σ showed a decrease in its expression similar to that of p53, p21 expression was significantly induced (Fig.6D). When we conducted ChIP assay using antibodies against H3Ac, we found significant loss of H3Ac occupancy in the p53 promoter region in NHK/H-Ras^G12V^ cells (Fig.6E), demonstrating that deacetylation of p53 may also play a role in inhibiting p53 expression in OIS.

## Discussion

In this report, we showed that the level of p53 diminishes during replicative senescence and OIS in NHKs *in vitro* as well as in epithelial layers of human oral mucosa by aging *in vivo*. We also showed that the reduction of p53 expression occurs at the transcriptional level and that this diminution is associated with histone deacetylation at the p53 promoter regions. Our study demonstrated that, unlike in fibroblasts, replicative senescence in NHKs is not caused by enhanced intracellular level of p53, as the level of p53 and its downstream gene products are progressively diminished during replicative senescence.

Despite the diminution of p53 mRNA and protein levels, pathways leading to p53 activation seem to be intact in senescent NHKs. Upon exogenous (e.g., radiations, carcinogens, etc.) or endogenous stresses (e.g., telomere shortening, etc.), p53 primarily undergoes posttranslational protein modifications, leading to increased protein level and enhanced transactivation activities (Lee & Gu, 2010). When treated with MMC to activate the DNA damage response, both young and senescent NHKs exhibited p53 activation (e.g., phosphorylation and transactivation activity; Fig.1F,G) although the magnitude of p53 activation was significantly reduced in senescent NHKs compared to that in younger cells. Such difference reflects a progressive decrease in the basal p53 protein level during replicative senescence and is consistent with previous finding that resistance to UV-induced apoptosis in senescent NHKs is associated with the lack of p53 activation (Chaturvedi *et al*., 2004).

In contrast to G_1_ cell cycle arrest induced by overexpression of p53 in fibroblasts, (Afshari *et al*., 1993; Dulić *et al*., 1993; Stein & Dulić, 1995; Wesierska-Gadek *et al*., 2005), senescent NHKs exhibited G_2_ cell cycle arrest (Fig.2A,B) suggesting that replicative senescence in NHKs mainly occurs independently of p53 expression status. However, it should also be noted that such observation may be due to mitotic slippage as senescent NHKs arrest with 4N DNA contents (Gandarillas & Freije, 2014). Role of cell cycle arrest during replicative senescence in NHKs should further be clarified.

Although the level p53 is generally known to be regulated at the posttranslational level via ubiquitin-dependent proteasomal degradation pathway, our study showed that such mode is not the primary regulatory mode of p53 diminution during replicative senescence in NHKs (Fig.3A,B). In line with our observation, it is noteworthy that senescent cells generally exhibit accumulation of abnormally synthesized or modified proteins, rather than rapid protein turnover, due to impaired protein degradation pathways (Cuervo & Dice, 1998). On the other hand, our data indicate that p53 mRNA level diminishes during replicative senescence (Fig.3D). Such diminution was not dependent on cell culture conditions or antibody selection (Figs S2, S4, and S5), indicating that our findings are less likely to be experimental artifacts.

Recent studies have revealed that p53 is transcriptionally regulated by epigenetic mechanisms (Su *et al*., 2009; Soto-Reyes & Recillas-Targa, 2010). These earlier studies found that the repression of p53 transcription was associated with the open chromatin conformation and not DNA hypermethylation, suggesting that histone modification may play a major role in the regulation of p53 expression. Consistent with these findings, our data showed that the p53 promoter methylation is not induced by replicative senescence (Figs4A–C and S6). Instead, chromatin remodeling of the p53 promoter was significantly associated with regulating p53 transcription as demonstrated by H3Ac depletion and, H3K27me3 enrichment to minor extent, at the p53 promoter regions in senescent NHKs (Fig.4D). An increase in the repressive histone marks and decrease in active marks during aging have been shown recently (Cheung *et al*., 2010; Wood *et al*., 2010). Therefore, we speculate that the progressive decrease in p53 transcription during organismal aging is mediated by chromatin condensation at the p53 locus, preventing the recruitment of transcriptional machinery at the promoter regions, consequently reducing the p53 gene expression.

During OIS, we also found that p53 expression diminishes and that histone deacetylation is also involved in p53 regulation (Fig.6). In fibroblasts, OIS has been shown to induce p53 level (Serrano *et al*., 1997); however, direct involvement of p53 in mediating OIS has been a subject of controversies. Takaoka *et al*. (2004) showed that H-Ras^G12V^ induced OIS in the presence of dominant-negative p53 mutants in esophageal keratinocytes, indicating that p53 is dispensable in H-Ras^G12V^-mediated senescence. Furthermore, the loss of p53 was also demonstrated in H-Ras^G12V^-induced OIS in primary mammary epithelial cells (Cipriano *et al*., 2011). These studies, along with our current findings, suggest that p53 does not play a primary role in inducing OIS and that the loss of p53 transcription may be a general tendency in cells undergoing senescence.

During replicative senescence in NHKs, expression of p53 target gene p21 was also diminished (Figs1, 2 and 3). Although p21 is known to increase significantly in senescence of human diploid fibroblasts (Noda *et al*., 1994), other study showed that p21 is not required for causing replicative senescence (Medcalf *et al*., 1996). In contrast, p21 expression during OIS in NHKs was significantly overexpressed (Fig.6). This finding is in line with previous observation in which p21 was also significantly induced in H-Ras^G12V^-mediated senescence in p53-deficient human mammary epithelial cells (Cipriano *et al*., 2011), suggesting that there is p53-independent and p21-dependent growth suppression in OIS.

Age-dependent increase in the cancer incidence is largely attributed to accumulation of genetic mutations. We previously reported that senescent NHKs exhibited increased frequency of errors in DNA repair mechanisms (Kang *et al*., 2005). By knocking down the p53 expression in NHKs, we demonstrated that increased error frequency is directly related to the loss of p53 expression (Fig.5). These data are consistent with previous *in vivo* findings in mouse models. For example, in transgenic mouse models, augmenting the endogenous p53 activity with a truncated form of p53 or an extra copy of wild-type, full-length p53 transgene enhanced resistance to spontaneous tumor development (García-Cao *et al*., 2002; Tyner *et al*., 2002). Although enhancing the p53 transcriptional activity revealed anticancer effects in both models, the mice with truncated form of p53 exhibited premature aging phenotype, whereas those with full-length p53 did not. Close examination showed that the p53 transgene was transcriptionally regulated under the endogenous p53 promoter which may have been subjected to epigenetic regulation during aging process. These data suggest that tight regulation of p53 expression level and its activity under its natural promoter may be important in balancing between the functions of tumor suppression and premature aging.

Our study showed age-dependent loss of p53 expression in normal oral epithelial tissues (Fig.1D,E). Significant p53-positive nuclear staining patterns were frequently reported in basal cell layers of oral and skin epithelium (Hausmann *et al*., 1998; Bortoluzzi *et al*., 2004; Abbas *et al*., 2007; Humayun & Prasad, 2011), suggesting that p53 expression in normal oral epithelium may play an important role in maintaining tissue homeostasis. On the other hands, p53 is generally considered to be absent in normal epithelial cells. It is noteworthy that many of these studies evaluated p53 expression in patients with cancer whereby normal status of p53 was assessed using tissues from the age-matched groups or the normal adjacent sites that are otherwise defined to be normal by histological means (Ren *et al*., 1996; Pontén *et al*., 1997; Cruz *et al*., 2002). As such, our finding of prominent p53-positive staining in relatively younger patient pools (e.g., below 40s) and a progressive decrease in its expression thereafter may partly explain the discrepancy between our findings and previous reports.

In conclusion, our study demonstrates that replicative senescence and OIS in keratinocytes are not induced by p53 but rather result from an intrinsic aging mechanism through which p53 transcription is directly suppressed possibly via histone deacetylation. Because p53 is a potent tumor suppressor known as the guardian of the genome, it is possible that senescent or aged cells lacking p53 are more vulnerable to an accumulation of genetic mutations and may be predisposed to undergo cancer transformation. Therefore, intervening epigenetic mechanisms of p53 to maintain the significant level of p53 throughout the lifespan during organismal aging may provide a therapeutic clue to prevent cancer development.

## Materials and methods

### Reagents and antibodies

MG132, actinomycin D, 5-aza-2′-deoxycytidine (5-aza-CdR), MMC, and zebularine (Zeb) were purchased from Sigma (St Louis, MO, USA). The following antibodies were purchased: P53 (DO-1), p16 (F-12), caspase-3 (H-277), β-actin (I-19), GAPDH (FL335), cyclin D1 (C-20), CDK6 (C21), cyclin A2 (C-19), CDK2 (M2), cyclin B1 (GNS1), and PCNA (PC10) from Santa Cruz Biotechnology (Santa Cruz, CA, USA); CDK1 (Ab-3) and p21 (EA10) from Calbiochem (San Diego, CA, USA); phospho-p53 at Ser15 (#9286), phospho-p53 at Ser46 (#2521), phosphor-Rb at Ser807/811 (#9308), Rb (#9309), and PUMA (#4976) from the Cell Signaling Technology, Inc. (Denver, MA, USA); p53 (Ab-2), H3K27me3 (07-449), and H3Ac (07-599) from EMD Millipore (Billerica, MA, USA); and p53 (DO-12) kindly provided by Dr. Borek Vojtesek (Masaryk Memorial Cancer Institute, Czech Republic). PG13-luciferase (wild-type p53 binding site) and MG15-luciferase (mutant p53 binding site) reporter vectors were kindly provided by Dr. Bert Vogelstein and Kenneth W. Kinzler (Johns Hopkins University, Baltimore, MD, USA).

### Cells and cell culture

Primary NHKs were obtained under the approval from Institutional Review Board (IRB# 04-060-04) as discarded tissues without patient identifiers from the normal patients who are undergoing routine dental procedures (e.g., gingivectomy for the crown-lengthening procedures). NHKs were prepared from keratinized oral epithelial tissues according to methods described elsewhere (Kim *et al*., 2010). Briefly, detached cells were seeded onto flasks and cultured in keratinocyte growth medium supplemented with a bullet kit (Lonza Biologicals Inc., Portsmouth, NH, USA) or EpiLife supplemented with HKGS (Invitrogen, Calsbad, CA, USA). These cells were serially subcultured until they senesced, and the cumulative population doublings and replication kinetics were determined based on the number of NHKs harvested at every passage.

### Retroviral vector construction and transduction of cells

The pSIREN-RetroQ vectors (BD Biosciences) without (EV) or with p53shRNA (p53sh) were used to prepare retroviruses. Also, pBabe and pBabe-H-Ras^G12V^ (Rangarajan *et al*., 2004; plasmid #12274; Addgene, Cambridge, MA, USA) were also used. Briefly, pSir or p53sh retroviral vectors were transfected into GP2-293 universal packaging cells (Clonetech, Mountain View, CA, USA) along with pVSV-G envelope plasmid using a calcium-phosphate transfection method. Two days after transfection, the virus supernatant was collected and concentrated by ultracentrifugation. The virus pellet was suspended in keratinocyte growth medium or EpiLife and was used for infection or stored at −80 °C for later use. NHKs were infected with retroviruses containing pSir, p53sh, pBabe, and pBabe-H-Ras^G12V^ in the presence of 6 μg mL^−1^ polybrene for 3 h. All of these viruses consistently gave more than 90% of infection efficiency (Kim *et al*., 2010). Drug selection of cells began at 48 h after infection with 1 μg mL^−1^ puromycin. The drug-resistant cells were maintained in subcultures as described above.

### Western blotting

Whole cell extracts from cultured cells were fractionated by SDS-PAGE and transferred to Immobilon protein membrane (Millipore). Immobilized membrane was incubated with primary antibodies and probed with the respective secondary antibodies conjugated with HRP. The signals were obtained using ChemiDoc XRS System (Bio-Rad, Hercules, CA, USA).

### Cell cycle analysis

Young and senescent NHKs were harvested and suspended in the hypotonic DNA staining buffer (3.4 mm sodium citrate, 0.3% Triton X, 20 μg mL^−1^ ribonuclease A and 100 μg mL^−1^ propidium iodide) for 30 min to 1 h at 4 °C and processed for the cell cycle analysis. The data were obtained using the flow cytometer in the UCLA Flow Cytometry Core Laboratory.

### Luciferase assay

Prior to transfection, a six-well plate with 5 × 10^2^ cells per well was plated and cultured for 24 h. The PG13-Luc vector containing 13 copies of the p53-binding consensus sequence cloned from the p21 promoter and MG-15-Luc vector containing the mutant p53-binding consensus sequence were kindly provided by Dr. Bert Vogelstein. These vectors (1 μg per well) were introduced into the cells using Effectene Transfection Reagent (Qiagen, Valencia, CA, USA). To control the differences in transfection efficiency, pRL-SV40 plasmid (10 ng per well) containing *Renilla* luciferase gene under SV40 enhancer/promoter was cotransfected into the cells. After transfection, cells were treated with mitomycin C (10 μm for 36 h. Cells were then harvested, and the luciferase activity was measured using the Dual Luciferase Reporter assay system (Promega Corporation, Madison, WI, USA) and the luminometer.

### RNA isolation and real-time quantitative RT–PCR

Total RNA was isolated from the cultured cells using RNeasy Plus Mini Kit (Qiagen, Chatsworth, CA, USA). DNA-free total RNA (5 μg) was dissolved in 15 μL DEPC-H_2_O, and the RT reaction was performed in first-strand buffer (Invitrogen) containing 300 U Superscript II (Invitrogen), 10 mm DTT, 0.5 μg random hexamer (Promega Corporation, Madison, WI, USA), and 125 μm dNTPs. The annealing reaction was carried out for 5 min at 65 °C, and cDNA synthesis was performed for 2 h at 37 °C, followed by incubation for 15 min at 70 °C to stop the enzyme reaction. The RT product was diluted with 70 μL H_2_O. The primers used for PCR amplification are listed in the Table S1 (Supporting information).

### Bisulfite sequencing assay

The methylation status of the p53 promoter was examined using EZ DNA Methylation Kit (Zymo Research, Irvine, CA, USA) according to the manufacturer’s instructions. Briefly, genomic DNA was isolated using DNeasy Blood and Tissue Kit (Qiagen Inc.). Genomic DNA was treated with CT conversion buffer containing sodium bisulfite to convert cytosine into uracil. Bisulfite-treated genomic DNA was PCR-amplified using p53 CpG island-specific primers (Table S2; Supporting information) designed by the MethPrimer program (Li & Dahiya, 2002). The resultant PCR-amplified fragments were cloned into TOPO TA cloning vector (Invitrogen), and 10 different clones were sequenced (Laragen Inc., Culver City, CA, USA) to compare the methylation status of the p53 promoter from young and senescent NHKs.

### Chromatin immunoprecipitation assay

Cells were fixed at room temperature for 10 min in the culture medium containing 1% formaldehyde, and ChIP assay was performed using the MAGnify ChIP System (Invitrogen) according to the manufacturer’s instructions. H3Ac and H3K27me3 antibodies were coupled to Dynabeads, and the antibody-coupled Dynabeads were incubated with the sheared chromatin. Chromatin–antibody–Dynabeads complexes were then washed with washing buffers, and the cross-links were reversed in the presence of proteinase K. The un-cross-linked DNA was purified using the DNA purification magnetic beads. The purified DNA fragments were amplified using primer sets that target the p53 promoter regions (Table S3; Supporting information).

### *In vitro* DNA end joining assay

DNA end joining assay was performed as described previously (Kang *et al*., 2005). Briefly, young and senescent NHKs from the same strain were collected, and the cells were lysed by incubating in CHAPS buffer for 30 min at 4 °C. The lysed cells were centrifuged at 8000 ***g*** for 10 min at 4 °C, and the supernatants were collected. The *in vitro* DNA end joining reaction was carried out by mixing the supernatants (5 μg) with ECoRI or EcoRV-treated linearized pCR2.1-TOPO plasmid (10 ng, Invitrogen) in the presence of 10% PEG and ligase buffer for 2 h at 37 °C. After reaction, PCR was performed using the M13 primers to amplify rejoined DNA. The PCR products were separated in 2% agarose gel electrophoresis.

To access the accuracy of the end joining activity, we cloned the PCR products from the ECoRI-linearized and ligated pCR2.1-TOPO plasmid into pcDNA3.1/V5-His TOPO plasmid (Invitrogen). The resulting ligated products were introduced into TOP10 cells (Invitrogen), and the single colony PCR was performed using the M13 primers (100 clones per samples). The PCR product was digested with EcoR1, and the digested products were electrophoresed in 2% agarose gels. The ECoRI-resistant PCR products were considered as abnormal end joining with sequence alterations.

### *De novo* mRNA synthesis assay

To label the newly synthesizing mRNA in young and senescent NHKs, we used Click-iT Nascent RNA Capture Kit (Invitrogen) according to the manufacturer’s instructions with minor modifications. Briefly, newly transcribing mRNAs were labeled by incubating young and senescent NHKs with 200 μm of 5-ethynyl uridine (EU) for 2 h. Cells were collected, and the nuclear mRNAs were isolated using Cytoplasmic and Nuclear RNA purification Kit (Norgen Biotek, Corp., Thorold, ON, Canada). The EU-labeled mRNAs were biotinylated and captured using Dynabeads MyOne Streptavidin T1 magnetic beads (Invitrogen). The purified mRNAs were subjected to cDNA synthesis and qRT–PCR.

### Immunohistochemical/IF staining

*In situ* p53 expression was determined in normal human oral epithelial samples obtained from the local dental offices according to the guidelines of the University of California at Los Angeles Institutional Review Board (IRB). NHOE was fixed, paraffin-embedded, and sectioned at 4 μm in the Translational Pathology Core Laboratory (TPCL) at UCLA. Slides were deparaffinized at 60 °C in oven for 30 min followed by rehydration in xylene and ethanol. The slides were then unmasked in citrate buffer (6 mm citric acid/34 mm sodium citrate/pH.6.0) above 95 °C for 25 min, and the endogenous peroxidase was blocked with 3% hydrogen peroxide (H_2_O_2_). The slides were incubated with 10% blocking buffer for 30 min, p53 or anti-mouse IgG (Vector Laboratories Inc., Burlingame, CA, USA) antibodies (1:100) in 3% blocking buffer for 90 min, secondary antibody (1:200) in 3% blocking buffer for 60 min, and HRP–avidin (1:1000) in PBST for 30 min. The slides were developed using DAB substrate kit (Vector Laboratories, Inc.).

For quantification, the representative field was randomly selected by a third person, and one thousand cells were counted for heavy staining (> 80%), moderate staining (40–80%), mild staining (5–40%), or no staining (< 5%). Only the heavy-staining cells were counted and plotted against the age.

For immunofluorescence (IF) staining, NHKs were plated and allowed for proliferation for 2 days. Cells were then fixed with 100% methanol for 15 min, washed with PBS for three times, and incubated with 6% BSA for 1 h followed by the primary p53 antibody (DO-1) incubation in IF solutions (6% BSA/0.6% Triton X-100 in PBS) for 1 h at 37 °C. The Alex Fluor 488 goat anti-mouse IgG (A-21202; Invitrogen) was incubated for 1 h, and the cells were mounted with ProLong Gold mounting medium (Invitrogen). Green fluorescence was observed and imaged using Olympus CKX41 fluorescence microscope (Center Valley, PA, USA).

## Statistical analysis

All experiments were performed in triplicate, and the measurements were expressed as mean ± SD. The numbers of p53 staining and ages were analyzed using simple linear regression. A two-way χ^2^ test was used to evaluate the mutation frequency from the *in vitro* DNA end joining assay. *p* < 0.05 was considered statistically significant.

## Supporting Information

**Fig. S1** Expression of p53 in normal human fibroblasts, IMR90 and WI38, during replicative senescence. **Fig. S2** p53 is high in early stages of primary culture. **Fig. S3** MG132 induces apoptotic responses in young but not in senescent NHKs. **Fig. S4** The loss of p53 expression during replicative senescence occurs independent of culture condition. **Fig. S5** The loss of p53 during replicative senescence in NHKs occurs in full length p53, FLp53, and the alternative splicing form, Δ40p53. **Fig. S6** Different demethylating agents do not re-express p53 in senescent NHKs. **Table S1** Primers for real-time qRT-PCR. **Table S2** Primers for bisulfite methylation assay. **Table S3** Primers for ChIP assay.
