# Supplementary material for: Regulation of p53 during senescence in normal human keratinocytes
Source: Aging Cell. 2015 Jul 1;14(5):838–46. doi: 10.1111/acel.12364 (PMC4568971; doi:10.1111/acel.12364)
Supplement: Supplementary file 1 [file acel0014-0838-sd1.docx]

Figure S1


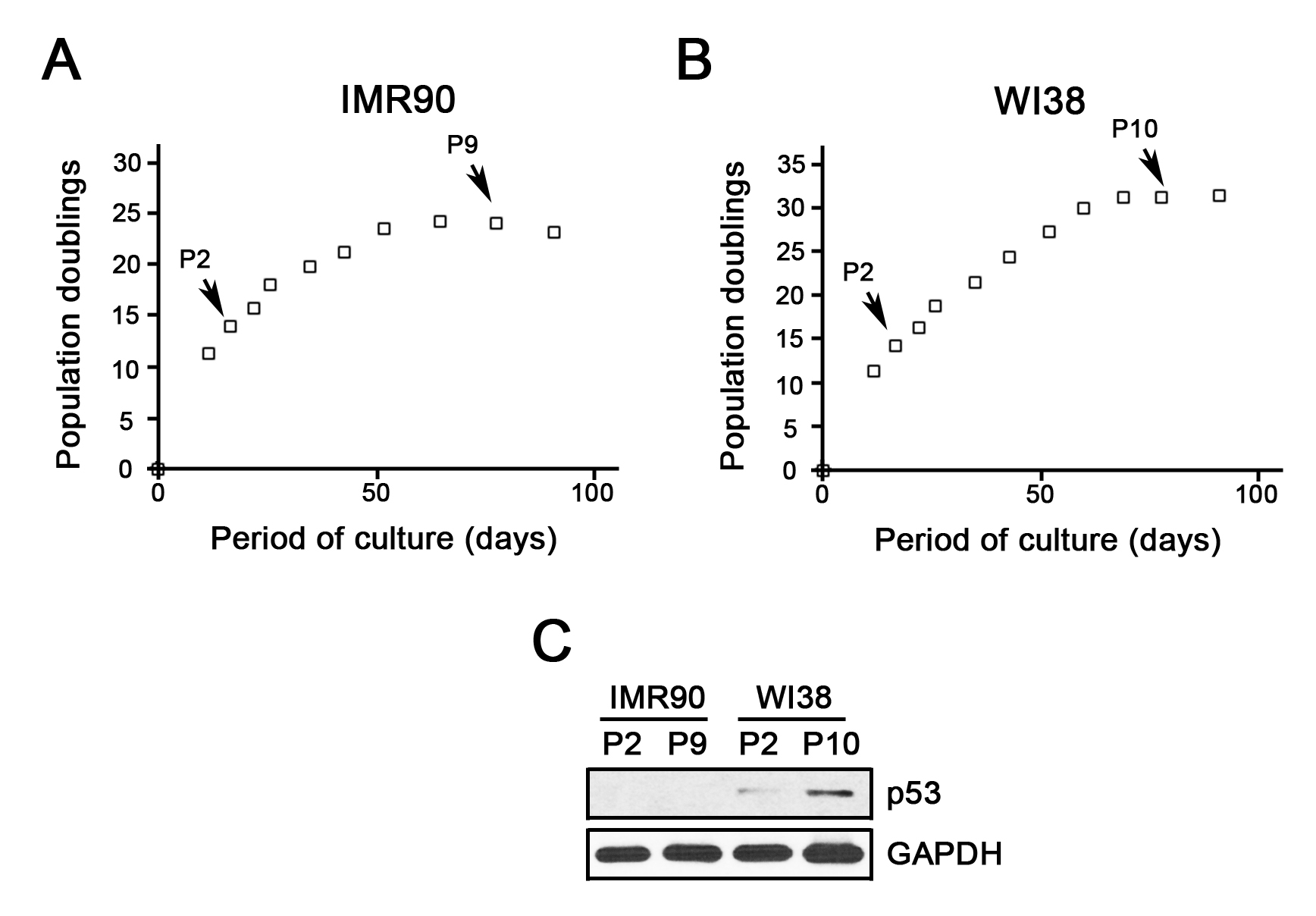


**Figure S1. Expression of p53 in normal human fibroblasts, IMR90 and WI38, during replicative senescence.** IMR90 (A) and WI38 (B) were serially subcultured until they reach senescence. (C) Cells from early (P2) and late stages (P9 or P10) were collected and subjected to Western blotting for p53 expression.

Figure S2


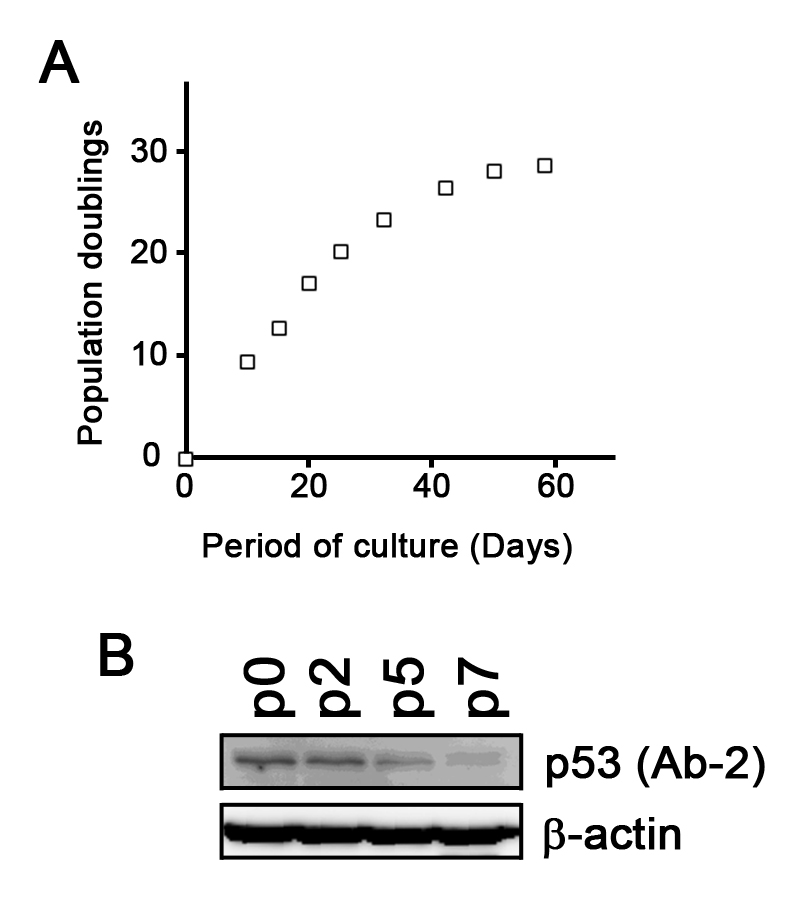


**Figure S2. p53 is high in early stages of primary culture.** (A) NHKs were newly prepared and PD curve was maintained. (B) NHKs from p0, p2, p5, and p7 were subjected to Western blotting against p53 using anti-p53 antibody (Ab-2).

Figure S3


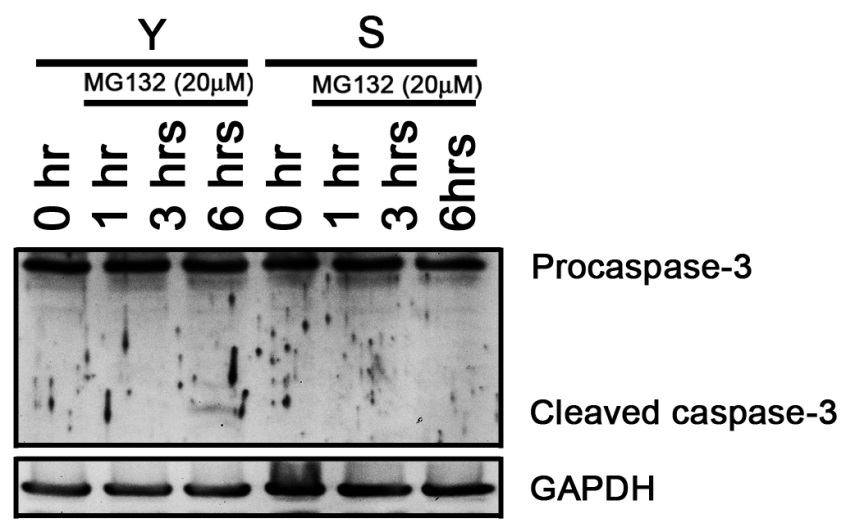


**Figure S3. MG132 induces apoptotic responses in young but not in senescent NHKs.** Actively proliferating young NHKs and senescent NHKs were treated with 20 µM MG132 for indicated hours. Cells were harvested, and Western blotting was performed against cleaved procaspase-3. GAPDH was used as a loading control.

Figure S4


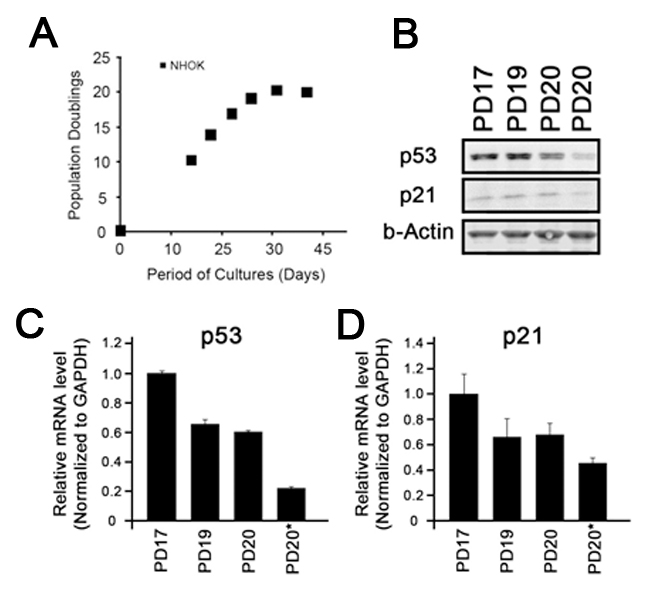


**Figure S4. The loss of p53 expression during replicative senescence occurs independent of culture condition.** (A) The primary NHKs were serially subcultured in KGM until they senesced, and the population doubling curve was obtained. (B) NHKs at the different PDs were subjected to Western blotting for the expressions of p53 and p21. β-Actin was used as a loading control. (C) The same cells were subjected to qRTpCR for p53 and p21. GAPDH was used to normalize.

Figure S5


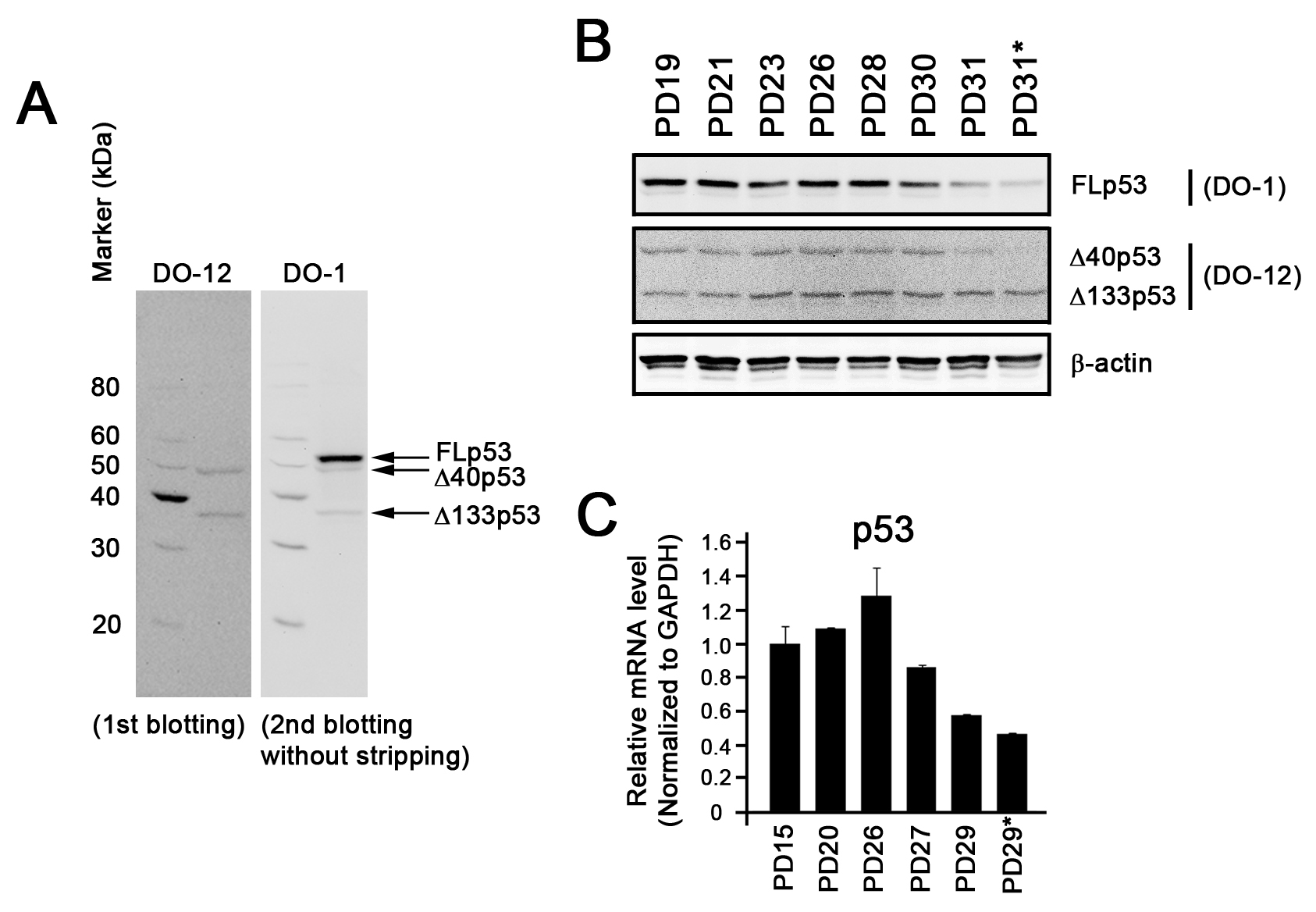


**Figure S5. The loss of p53 during replicative senescence in NHKs occurs in full length p53, FLp53, and the alternative splicing form, Δ40p53.** (A) Western blotting of p53 in actively proliferating in NHKs using two different clones, DO-12 and DO-1. (B) Serially subcultured NHKs were screened for the full-length p53 (FLp53) using α-p53 antibody (DO-1) and different alternative splicing forms, Δ40p53 and Δ133p53 using α-p53 antibody (DO-12). (C) Using a primer set that recognize the central region of p53 mRNA, qRT-PCR was performed. GAPDH was used for normalization.

Figure S6


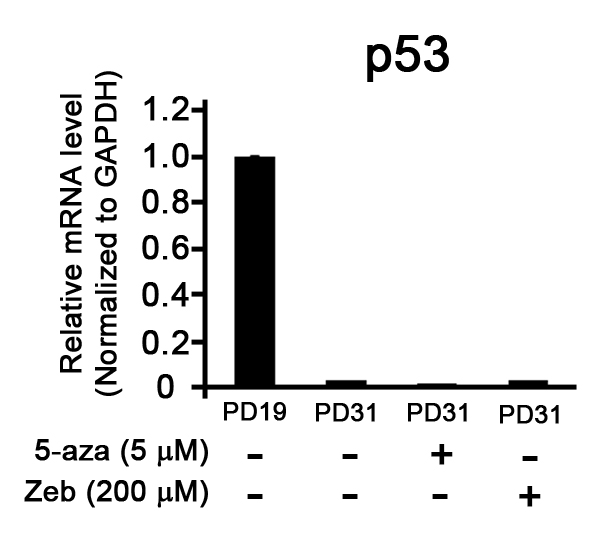


**Figure S6. Different demethylating agents do not re-express p53 in senescent NHKs.** Senescent NHKs were treated with 5-aza-CdR (5 µM) for 5 days or zebularine (200 µM) for 2 days and subjected to qRT-PCR to examine p53 expression. Young NHKs were included as a control. p53 mRNA expression was normalized to those of GAPDH.

Table S1: Primers for real-time qRT-PCR

| Name | Forward | Reverse |
| --- | --- | --- |
| p53 (5' region) | cagccaagtctgtgacttgcacgtac | actgcttgtagatggccatg |
| p53 (Central region) | tagtgtggtggtgccctatg | cacatgtagttgtagtggatggtg |
| p21 | ACAGCAGAGGAAGACCATGTGGACC | CGTTTTCGACCCTGAGAGTCTCCAG |
| P16 | GACTCTGGAGGACGAAGTTTGCAGG | GTCGCCAGGAGGAGGTCTGTGATTA |
| GADD45 | ggagagcagaagaccgaaag | tgactcagggctttgctga |
| 14-3-3σ | ccgaggtgcgtgagtacc | tgatgaggtggctgtcca |
| H-Ras | ggcatcccctacatcgaga | ctcacgcaccaacgtgtaga |
| PCNA | tggagaacttggaaatggaaa | gaactggttcattcatctctatgg |
| GAPDH | tccactggcgtcttcacc | ggcagagatgatgaccctttt |

Table S2: Primers for bisulfite methylation assay

| Name | Region | Forward | Reverse |
| --- | --- | --- | --- |
| CpG islands #1 | -1069 to -821 | TTTTTTTTGGGAGTAGGTAGAAGATT | AAAAACCCTAAAACTTAATAAAAAC |
| CpG islands #2 | 35 to 140 | GTTTAGGGAGTAGGTAGTTGTTGG | CAAAAAAAACTCATCAAATTCAATC |

Table S3: Primers for ChIP assay

| Name | Region | Forward | Reverse |
| --- | --- | --- | --- |
| Region #1 | -1100 bp | gaggaagcacagcggagat | cgtcggatccctgagaact |
| Region #2 | -100bp | gcccttacttgtcatggcga | ggctctagacttttgagaagc |
| Region #3 | Exon 1 | gctcaagactggcgctaaaa | gtcaccgtcgtggaaagc |
| Region #4 | 200bp | aggaccatccgaactcaaag | tcaaggttccccaaagctc |
| Region #5 | 4000bp | gcctgtaatcccagcacttt | tcaggctggtctcaaactcc |
